# Carnitine-acylcarnitine translocase deficiency: a case report with autopsy

**DOI:** 10.4322/acr.2024.483

**Published:** 2024-04-04

**Authors:** Chennakeshava Thunga, Suvradeep Mitra, Devi Dayal, Sadhna Lal

**Affiliations:** 1 Post Graduate Institute of Medical Education and Research (PGIMER), Department of Pediatric Gastroenterology, Hepatology, and Nutrition, Chandigarh, India; 2 Post Graduate Institute of Medical Education and Research (PGIMER), Department of Histopathology, Chandigarh, India; 3 Post Graduate Institute of Medical Education and Research (PGIMER), Department of Pediatrics, Endocrinology and Diabetes Unit, Chandigarh, India

**Keywords:** Autopsy, Fatty liver, Pathology

## Abstract

Fatty acid oxidation defects are a heterogeneous group of disorders related to the mitochondrial fatty acid oxidation pathway. Carnitine acylcarnitine translocase (CACT) is an enzyme responsible for the unidirectional transport of acylcarnitine across the inner mitochondrial membrane. This enzyme plays a crucial role in the oxidation of fatty acids. The autopsy pathology of the CACT deficiency is described in only a few cases. We describe the autopsy pathology of a child with CACT deficiency dominantly in the form of microvesicular steatosis of the hepatocytes, renal proximal tubular epithelia, cardiac myocytes, and rhabdomyocytes. The diagnosis was further confirmed on whole exome sequencing with compound heterozygous variants in the exon 1 (c.82G>T, p.Gly28Cys; likely pathogenic) and exon 5 (c.535G>A, p.Asp179Asn; uncertain significance) of the *SLC25A20* gene. This case elucidates the histopathology of the liver and the detailed autopsy of a case of CACT deficiency from India.

## INTRODUCTION

Fatty acid oxidation defects (FAOD) are a rare and heterogeneous group of inborn errors of metabolism related to defects in the mitochondrial fatty acid oxidation pathway.^1,2^ Carnitine acylcarnitine translocase (CACT) deficiency is a subtype of FAOD that causes severe non-ketotic hypoglycemia, hyperammonemia, arrhythmias, and sometimes culminates in a sudden unexpected death in infancy.^3-5^ We present herein the biochemical, histopathological, ultrastructural, and genetic data of a child with CACT deficiency with an affected elder sibling who underwent an autopsy.

## CASE REPORT

A 22-month-old girl, born to a non-consanguineously married couple, developed significant lethargy and generalized tonic-clonic seizures one week into a febrile illness due to an upper respiratory tract disease. She was referred to our center on the 7^th^ day of illness. Diagnostic investigations conducted at the referring hospital revealed hypoglycemia, hyperammonemia, normal lactate, and raised triglycerides. She had required a glucose infusion rate (GIR) of 6 milligrams/kilogram/minute.

The baby was afebrile, obtunded, and had acidotic breathing. Her anthropometry was normal (Weight: -0.06Z; Height: +1.5Z). She had mild pallor, was anicteric, had a massive firm hepatomegaly, and had a Glasgow coma scale (GCS) of E3M1V4. She was hypoglycemic (random blood sugar of 24mg/dL; normal range: 70-100 mg/dL), had metabolic acidosis (pH: 7.32; normal range: 7.35-7.45, bicarbonate: 14.3 mEq/L; normal range: 22-29 mEq/L). The ammonia was elevated (at admission: 249 μmol/L and terminally: 433.5 μmol/L; normal range: 11-32 μmol/L), low blood ketones during hypoglycemia (0.7mmol/L; normal range: <0.6mmol/L), normal lactate (11.4 mg/dL; normal range: 4.5-20 mg/dL), elevated total leucocyte count with neutrophilia (12,580 cells/μL, 63% neutrophils; normal range: 4000-11000/μL), elevated triglycerides (294 mg/dL; normal range: 50-75 mg/dL), raised creatinine (1.25 mg/dL; normal range: 0.3-0.5 mg/dL), and hyperuricemia (serum uric acid 13.7 mg/dL; normal range: 2.4-5.4 mg/dL). Her liver function test (LFT) revealed mild hyperbilirubinemia (total/ direct bilirubin: 2.21/1 mg/dL; normal range: 0.3-1.2/ 0.01-0.1 mg/dL), elevated AST (at admission: 329 IU/L and terminal: 2157 IU/L; normal range: 0-35 IU/L), elevated ALT (at admission: 150 IU/L and terminal: 934 IU/L; normal range: 0-35 IU/L), normal GGT (21 IU/L; normal range: 0-30 IU/L), elevated INR (1.5; normal range: 0.8-1.2), and near-normal synthetic function (total protein/ albumin: 4.7/ 3.5 g/dL; normal range: 5.5-8.0/ 3.5-5.5 g/dL).

The baby had suffered an episode of hypoglycemia needing intravenous correction on day 2 after birth and intercurrent acute gastroenteritis needing hospitalization (in another institute, details not available) for a week at 10 months of age. She was developmentally normal.

The child was started on glucose infusion (glucose infusion rate (GIR) at 6mg/kg/min), empirical intravenous antibiotics, and antiepileptics. Sodium benzoate and multivitamin cocktail were started for hyperammonemia and metabolic crisis. She was mechanically ventilated, given poor sensorium and breathing efforts. She developed right-sided pneumothorax and, subsequently, hypotension on the 30^th^ hour of admission (8^th^ day of illness). Her hypotension persisted even after draining pneumothorax. She developed multiple cardiac arrests and ultimately succumbed to her illness after 34 hours of admission at our institute.

## AUTOPSY FINDINGS

**Figure 1:**
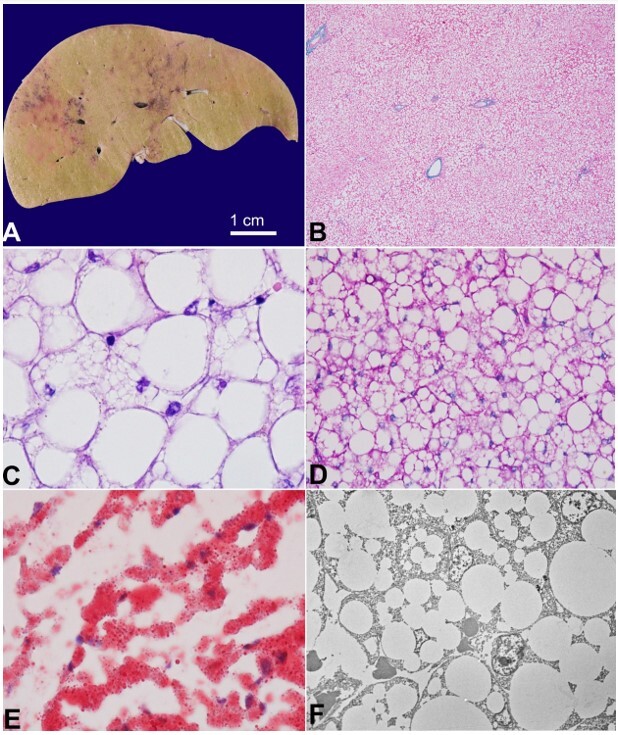
Gross and histomorphology of the liver: Yellowish and greasy cut surface of the liver with patchy areas of congestion (**A**); diffuse steatosis of the liver on microscopy with maintained lobular architecture (Masson trichrome, 40x, **B**), Microvesicular fat pushed to the periphery by the macrovesicular fat within the hepatocytes (Haematoxylin and eosin, 1000x, **C**); PAS stain did not highlight the vacuoles (400x, **D**); Oil red O highlighting intrahepatocytic fat (1000x, **E**); Transmission electron microscopy highlighting intrahepatocytic fat and lack of glycogen rosettes (Uranyl acetate and lead citrate, **F**).

A complete autopsy was performed within 6 hours of the death. The serous cavities were normal. Pneumothorax was not present at the time of the autopsy. The liver was heavy for the age and weighed 920 g (normal weight: 430-450 g). The cut surface was greasy and yellow, although there was no nodularity, capsular wrinkling, or space-occupying lesions (Figure 1A). Patchy areas of congestion were also noted. Microscopy highlighted maintained lobular hepatic architecture with diffuse steatosis involving almost all hepatocytes (~100% hepatocytes) (Figure 1B). The steatosis was both macrovesicular (~100% hepatocytes) and microvesicular, with the microvesicular fat being obscured by the macrovesicular fat. Thus, many hepatocytes showed microvesicular fatty change at the periphery of the cell. In contrast, the central part of the cell showed a single large fat globule indenting the nuclei and pushing it to the periphery (Figure 1C). No ballooned hepatocytes were seen. The hepatocytes showed no glycogen excess (PAS-negative) (Figure 1D), and the portal tracts were normal. Centrizonal sinusoidal dilatation, congestion, and patchy hemorrhagic necrosis (likely preterminal) were noted. No inflammation, portal or perisinusoidal fibrosis, or cholangiopathy was seen. The intracellular fat was stained by oil red O stain (Sigma-Aldrich) (Figure 1E). Transmission electron microscopy of the formalin-fixed liver tissue confirmed the presence of fat and ruled out any glycogen excess or glycogenic rosettes (Figure 1F).

**Figure 2:**
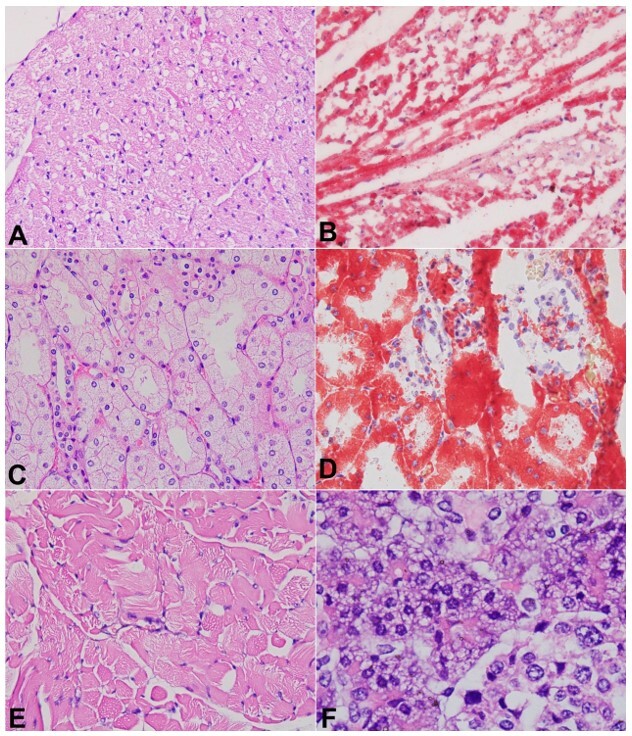
Histomorphology of the cardiac muscle, renal proximal tubular epithelia, skeletal muscle, and pancreatic exocrine acini: Cardiac myocytes containing multiple microvesicular fat (Haematoxylin and eosin, 400x, **A**) highlighted by oil red O stain (400x, **B**); renal proximal tubular epithelia containing multiple microvesicular fat giving it a bubbly appearance (Haematoxylin and eosin, 400x, **C**) highlighted by oil red O stain (400x, **D**); Occasional microvesicular changes noted within skeletal muscle myocytes (Haematoxylin and eosin, 400x, **E**) and pancreatic exocrine acini (Haematoxylin and eosin, 1000x, **F**).

The mitochondria appeared to be normal in size and shape. The internal structures of the mitochondria could not be commented on due to autopsy-related degenerative changes and formalin fixation. Similar microvesicular steatosis was also noted in the cardiac myocytes (Figures 2A and 2B), proximal convoluted tubules of the kidneys (Figures 2C and 2D), occasional skeletal muscle cells (Figure 2E), and pancreatic exocrine acini (Figure 2F), confirmed by oil red O stain performed on the cryosections of these tissues.

The lungs weighed 200 g (normal weight: 150-170 g). The pleural surface showed patchy basal dullness, and both lungs showed basal consolidation. Microscopy revealed lobar pneumonia involving bilateral lower lobes with numerous Gram-negative bacilli. A few foci showed intra-alveolar hemorrhage. The remaining organs, including the brain, spleen, thymus, lymph nodes, bone marrow, thyroid, adrenal, endocrine pancreas, gastrointestinal, and genitourinary tracts, were grossly and microscopically unremarkable.

Whole exome sequencing from blood on Illumina sequencing platform (average sequencing depth: 250) revealed compound heterozygous variants in the exon 1 (c.82G>T, p.Gly28Cys; likely pathogenic) and exon 5 (c.535G>A, p.Asp179Asn; uncertain significance) of the *SLC25A20* gene (carnitine-acylcarnitine translocase deficiency).

A final autopsy diagnosis of fatty acid oxidation defect caused by carnitine-acylcarnitine translocase deficiency due to compound heterozygosity of the *SLC25A20* gene complicated by lobar pneumonia caused by Gram-negative bacilli was rendered. Although the genetic testing highlighted a compound heterozygous state of a likely pathogenic variant and a VUS (variant of uncertain significance) of the *SLC25A20* gene, the combination of clinical, biochemical, and histopathological features suggested a fatty acid oxidation defect to be the most likely possibility.

## HISTORY AND HISTOPATHOLOGY OF THE LIVER BIOPSY IN THE ELDER SIBLING

**Figure 3:**
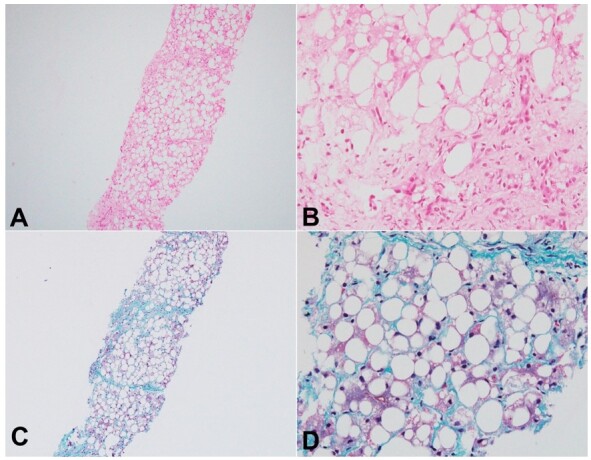
Histomorphology of liver biopsy in the elder sibling: Diffuse hepatocellular steatosis (Haematoxylin and eosin, 100x, **A**); a few hepatocytes showed microvesicular fat (Haematoxylin and eosin, 400x, **B**); incomplete nodule formation and bridging septa (Masson trichrome, 100x, **C**); occasional foci showing pericellular fibrosis (Masson trichrome, 400x, **D**).

There was a significant family history of a similarly affected elder male sibling (two years back) who had suffered a cardiac arrest with ventricular arrhythmia, severe hypoglycemia (7mg%), hyperammonemia, severe hyperkalemia (Potassium of 9 mEq/L) and renal dysfunction on 3^rd^ day of life. He survived this and was detected to have a firm hepatomegaly at 4 months of his life. He succumbed after a brief illness at 10 months of age. The liver biopsy of the previous male sibling (performed during the fourth month of life) was reviewed, which showed similar features of the hepatocytes However, there was portal, portoseptal, and perisinusoidal fibrosis with occasional incomplete nodule formation (Figure 3).

## DISCUSSION

Fatty acid oxidation defects (FAOD) are a heterogeneous group of inborn errors of metabolism, some of which may present as sudden, unexpected death of infancy (SUDI). It may account for up to 5% of SUDIs, and affected children have a high mortality before reaching 5 years of age.^1,2^ The pathogenesis of this group of disorders is related to the mitochondrial oxidation of fatty acids, which is a crucial energy source, especially in newborns, as they have low glycogen stores. Besides, the mitochondrial oxidation pathway of the fatty acids is also important during prolonged starvation. The adipose tissue’s fatty acids need to be mobilized during these periods of starvation/ following birth as the child is deprived of the continuous food supply, and the body relies on the fat as the main energy source. These mobilized fatty acids are then oxidized in the mitochondria, generating ketone bodies. The FAODs related to various enzyme mutations prevent the fatty acids from being available during the initial days after birth, during prolonged starvation, and during exercise, causing energy deprivation to the child. Toxic Acyl CoA esters, acylcarnitines/acylglycines accumulate in tissues, and secondary carnitine depletion is common. Cardiac and skeletal muscle and renal tubules derive much of their energy at all times from long-chain fatty acid oxidation and are thus commonly involved in their defects.^3-6^

**Figure 4:**
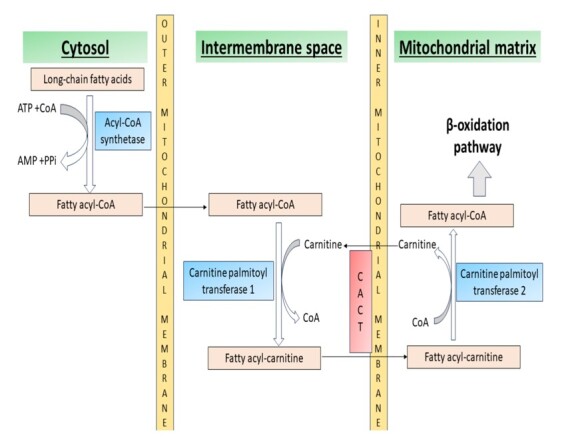
Schematic diagram highlighting the mechanism by which long-chain fatty acids enter the mitochondria.

Carnitine-acylcarnitine translocase (CACT) is an essential enzyme in the fatty acid oxidation pathway. This enzyme is responsible for the unidirectional transport of the acylcarnitine across the inner mitochondrial membrane. Figure 4 depicts the schematic diagram highlighting the mechanism by which long-chain fatty acids enter the mitochondria along with the role of CACT.^7^

It makes acylcarnitine available inside the mitochondria to transfer of the acyl group to coenzyme A for further propagation of the fatty acid oxidation pathway. The CACT belongs to a family of mitochondrial carrier proteins known as SLC25 and is coded by *SLC25A20* gene located on chromosome 3p21.31. CACT deficiency is an autosomal recessive uncommon subtype of FAOD with less than 75 cases reported in the literature.^3-6^ It behaves like long-chain fatty acid oxidation defects (LCHAD). We describe the clinical, histopathological, and sequencing data of a case of CACT deficiency in this index study. It is worthwhile to mention the deficiencies of two related enzymes, namely carnitine palmitoyltransferase 1 (CPT1) and carnitine palmitoyltransferase 2 (CPT2), that can present with similar clinical manifestations and are components of the same pathway (Figure 4). CPT1 and CPT2 are located at the outer and inner mitochondrial membranes, catalyzing the conversion of fatty acyl-CoA to fatty acyl-carnitine and vice versa at their respective sites. The CPT1 enzyme has liver and muscle isoforms, and the deficiency of the former isoform characteristically presents with recurrent hypoketotic hypoglycemia. The infantile form of CPT2 deficiency also presents with hypoketotic hypoglycemia and SUDI due to cardiac damage.^8^

The clinical features of CACT deficiency consist of hypo/non-ketotic hypoglycemia, hyperammonemia, cardiomyopathy, sudden cardiac death, liver dysfunction, renal dysfunction, muscle weakness, and severe neurological dysfunction. Intercurrent respiratory infections can be a cause of morbidity and mortality with precipitation of hypoglycemic episodes. The index case showed non-ketotic hypoglycemia refractory to glucose infusion, hyperammonemia, firm hepatomegaly, liver dysfunction, and renal dysfunction.^3-5^ These features, along with a similarly affected sibling, point towards the diagnosis of FAOD, likely caused by CACT deficiency, which was confirmed by clinical exome sequencing.

The histopathology in the index case highlighted microvesicular steatosis in the organs rich in mitochondria, including the liver, proximal tubular epithelia, cardiac myocytes, and skeletal muscle cells. The histomorphology of the liver shows diffuse microvesicular steatosis obscured by macrovesicular steatosis in the absence of any significant portal or lobular fibrosis and/or inflammation. The presence of microvesicular steatosis can be confirmed by oil red O stain as well as transmission electron microscopy (TEM). Besides, the TEM also provides an idea about the glycogen and subcellular organelles, including mitochondria, endoplasmic reticulum, and Golgi apparatus. Glycogen storage disease can present with diffuse macrovesicular steatosis. However, the hepatocytes without significant fatty change may show the typical plant-cell-like morphology with PAS-positive and diastase-sensitive glycogen. Numerous other conditions give rise to steatosis in pediatric liver, including various inborn errors of metabolism, malnutrition, pediatric fatty liver disease, Reye's syndrome, drug-induced liver injury, and acute hepatitis.^9^ The fatty changes in the hepatocytes, proximal tubular epithelia, skeletal, and cardiac myocytes are responsible for the dysfunction of the respective organs, contributing to morbidity and mortality. Similar findings were also demonstrated in a previously documented autopsy case caused by CACT deficiency.^10^ The diffuse fatty change corroborates with the firm hepatomegaly and the increase in the weight of the liver. Despite extensive literature search, we did not find any mention of microvesicular steatosis of exocrine pancreatic acini, and it appears to be a novel finding in the index case. Microvesicular steatosis within the exocrine pancreatic acini (“intra-acinar” fat) has been documented in adults in relation to various fatty liver diseases, including metabolic dysfunction and ethanol consumption.^11^ Our patient’s elder sibling showed portal, portoseptal, perisinusoidal, and occasional portoportal bridging fibrosis with occasional nodule formation in the antemortem liver biopsy at the fourth month of life. In such an early stage, advanced fibrosis is also unusual for CACT deficiency, and the cause remained elusive short of a genetic analysis. Costa et al.^12^ had previously described a similar defect in the exon 1 (c.82G>T, p.Gly28Cys) with a mild phenotype contrary to the histopathology of the elder sibling. Another interesting feature of this case was the marked hyperuricemia, which has not been highlighted in the literature. A possible cause might be rhabdomyolysis, which is known to be associated with the metabolic crisis in this disorder and renal failure. Unfortunately, we lack histologic proof of the same, though indirect evidence may be the documented hyperkalemia during the metabolic crisis of the elder sibling.

The treatment of CACT deficiency requires glucose infusion, prevention of fasting state by administering frequent meals, carnitine supplementation, and dietary modification in the form of a high-carbohydrate/low-fat diet rich in medium-chain triglycerides.^3-5^ Prompt treatment of intercurrent infections is important as they cause precipitation of hypoglycemic episodes. A few children can be refractory to the glucose infusion, similar to the index case. The mortality rate is as high as 65% due to cardiomyopathy, cardiac arrhythmia, heart block, or other cardiac dysfunction during the first year of life leading to SUDI.^3^ Recently, the supplementation with Triheptanoin, a seven-carbon triglyceride, has shown promising results in the treatment of CACT and LCHAD.^12,13^

## CONCLUSION

We present an autopsy case of CACT deficiency with a compound heterozygous state with clinical, biochemical, histopathological (autopsy pathology) details, ultrastructural, and genetic data. This is an uncommon case and highlights the need for prompt antemortem diagnosis for the adequate management of this near-fatal defect. The index case highlights the clinical and pathological spectrum of CACT deficiency, correlating the clinical, biochemical, and pathological features.
